# Microbiota-derived lactate promotes hematopoiesis and erythropoiesis by inducing stem cell factor production from leptin receptor+ niche cells

**DOI:** 10.1038/s12276-021-00667-y

**Published:** 2021-09-09

**Authors:** Yong-Soo Lee, Tae-Young Kim, Yeji Kim, Seungil Kim, Su-Hyun Lee, Sang-Uk Seo, Bo O. Zhou, O. Eunju, Kwang Soon Kim, Mi-Na Kweon

**Affiliations:** 1 Mucosal Immunology Laboratory, Department of Convergence Medicine, Asan Medical Center, University of Ulsan College of Medicine, Seoul, Republic of Korea; 2 Department of Microbiology, School of Medicine, The Catholic University of Korea, Seoul, Republic of Korea; 3 Shanghai Institute of Biochemistry and Cell Biology, Chinese Academy of Sciences, Shanghai, China; 4 Division of Integrative Biosciences and Biotechnology, Pohang University of Science and Technology, Pohang, Republic of Korea

**Keywords:** Immunology, Stem-cell research

## Abstract

Although functional interplay between intestinal microbiota and distant sites beyond the gut has been identified, the influence of microbiota-derived metabolites on hematopoietic stem cells (HSCs) remains unclear. This study investigated the role of microbiota-derived lactate in hematopoiesis using mice deficient in G-protein-coupled receptor (Gpr) 81 (Gpr81^−^^/−^), an established lactate receptor. We detected significant depletion of total HSCs in the bone marrow (BM) of Gpr81^−/−^ mice compared with heterogenic (Gpr81^+/−^) mice in a steady state. Notably, the expression levels of stem cell factor (SCF), which is required for the proliferation of HSCs, decreased significantly in leptin receptor-expressing (LepR^+^) mesenchymal stromal cells (MSCs) around the sinusoidal vessels of the BM from Gpr81^−/−^ mice compared with Gpr81^+/−^ mice. Hematopoietic recovery and activation of BM niche cells after irradiation or busulfan treatment also required Gpr81 signals. Oral administration of lactic acid-producing bacteria (LAB) activated SCF secretion from LepR^+^ BM MSCs and subsequently accelerated hematopoiesis and erythropoiesis. Most importantly, LAB feeding accelerated the self-renewal of HSCs in germ-free mice. These results suggest that microbiota-derived lactate stimulates SCF secretion by LepR^+^ BM MSCs and subsequently activates hematopoiesis and erythropoiesis in a Gpr81-dependent manner.

## Blood cell formation: Support from gut bacteria

Lactic acid produced by microbes in the gut has been implicated in supporting the production of blood cells, suggesting oral administration of lactic acid-producing bacteria might be useful for treating blood disorders, including anemia. Researchers in South Korea and China, led by Mi-Na Kweon at the University of Ulsan in Seoul, explored the significance of lactic acid using mice deficient in the gene for a protein receptor that allows lactic acid to influence various cellular processes. Without the benefit of this receptor the levels of blood cell-forming stem cells in bone marrow were reduced. The normal effect of lactic acid was linked to production of a protein called stem cell factor in specific cells. Oral administration of lactic acid-producing bacteria restored blood cell formation, indicating its therapeutic potential.

## Introduction

The mammalian intestine is a hub for commensal microbiota, which are important modulators of host physiology, metabolism, and immunity^1–3^. Recent studies have highlighted the functional interplay between intestinal microbiota and the host immune system beyond the gut, wherein intestinal microbiota-derived metabolites can likely impact disease pathophysiology at distant sites, including the lungs, liver, pancreas, skin, and bone marrow (BM)^4–8^. Additionally, research has shown that microbiota-derived short-chain fatty acids (SCFAs) can regulate osteoclast differentiation through metabolic reprogramming^8^. Furthermore, germ-free (GF) mice and antibiotic treatments have profoundly altered cellularity in the BM^9–13^. A suggested mechanism for this functional interplay is that microbiota-derived metabolites found in circulating blood indirectly affect target organs by modulating hematopoiesis at the BM.

The intestinal microbiota produces SCFAs (i.e., acetate, butyrate, and propionate), which are key factors required to maintain host metabolism and immunity^14–17^. G-protein-coupled receptors (Gprs) are well-known counterparts of SCFAs and play an indispensable role in metabolic and immunologic functions^14,15,18^. Exacerbation of inflammation in various disease models, such as colitis, arthritis, and asthma in Gpr43^−/−^ mice, suggests that SCFA-Gpr43 interactions regulate inflammatory responses^14^. Additionally, Gpr120, a receptor for long-chain fatty acids (i.e., ω-3 fatty acids), exerts potent anti-inflammatory effects on macrophages in vitro and in obese mice in vivo^19^. In particular, previous studies found that Gpr81 is a specific receptor for lactate^20–22^. We recently found that Gpr81-lactate interactions affect Wnt3 expression through Paneth and intestinal stromal cells, which consequently regulate the proliferation and differentiation of Lgr5^+^ intestinal stem cells^23^.

BM contains multipotential hematopoietic stem cells (HSCs) and progenitors, the mother cells capable of differentiating into all myeloid and erythroid lineages. In the perivascular niche, HSCs and progenitor cells are maintained by mesenchymal stromal cells (MSCs) and endothelial cells^24,25^. As primary niche cells, MSCs are located in the arterioles and sinusoids of the BM and express nerve/glial antigen (NG2) and leptin receptor (LepR), which regulate HSC quiescence and proliferation^26–28^. Approximately 80% of dividing HSCs and nondividing HSCs are present in sinusoidal blood vessels in the BM^29,30^. Furthermore, a recent study demonstrated that the differentiation fates of lymphoid, myeloid, and erythroid lineages and maintenance of HSCs through self-renewal are determined by the expression of stem cell factor (SCF) and SDF-1α cytokines by LepR^+^ MSCs^31,32^. Although metabolites from intestinal microbiota are expected to help maintain HSCs, less is known about their effects on BM niche cells and hematopoietic fate.

In this study, we investigated the effects of microbiota-derived lactate that passes through the systemic circulation to reach the BM and activates SCF expression of LepR^+^ cells in sinusoidal blood vessels of the BM in a Gpr81-dependent manner. We found that microbiota-derived lactate is critically associated with the proliferation of HSCs and further maintenance of hematopoiesis and erythropoiesis.

## Materials and methods

### Mice

Animal experiments were performed with adult 6- to 16-week-old mice with equal numbers of males and females. All mice were provided sterile pellet food and water ad libitum. C57BL/6N mice were purchased from Orient Bio (South Korea), and Gpr81^−/−^ mice were generated as described previously^23^. CD45.1^+^ (B6. SJL-Ptprc^a^ Pepc^b^/BoyJ), LepR-cre (B6.129(Cg)- LepR^tm2(cre)Rck^/J), and tdTomato-loxP (B6. Cg-Gt(ROSA)^26Sortm9(CAG-tdTomato)Hze^/J) mice were purchased from Jackson Laboratory and backcrossed in house. All animal experiments were approved by the Institutional Animal Care and Use Committee of the Asan Biomedical Research Center (Approval No: PN 2018-12-288) and conducted in compliance with regulatory guidelines. Mice were maintained under specific pathogen-free conditions at the Asan Biomedical Research Center (Seoul, South Korea), and GF mice were maintained in the animal facility at POSTECH (Pohang, South Korea). All animal experiments were performed under anesthesia with a mixture of ketamine (100 mg/kg BW) and xylazine (20 mg/kg BW).

### Purification of BM cells

BM plugs were flushed gently from the marrow cavity of two femurs and two tibias using a syringe fitted with a 23-gauge needle containing cold phosphate-buffered saline (PBS) to isolate BM mononuclear cells. Flushed cells were then gently drawn up into and expelled from a 26-gauge needle to dissociate clumps. Next, cells were filtered through a 40-µm strainer (BD Falcon), and recovered total BM cells were counted. Intact BM plugs from two femurs and two tibias were enzymatically digested in prewarmed digestion solution (DNase I [100 µg/mL, Roche], Liberase^DL^ [250 µg/mL, Roche] in HBSS plus Ca^2+^and Mg^2+^) and incubated at 37 °C for 30 min with gentle shaking (~120 r.p.m.) to isolate MSCs. After weak vortexing for 20 s, cells were allowed to settle for ~3 min, after which the supernatant was transferred to another tube on ice. The settled BM plugs were enzymatically digested repeatedly two times, as previously described. The collected cells were then counted with a 100-µm strainer (BD Falcon).

### FACS analysis

Total BM cells were collected from two femurs and two tibias and flushed gently to analyze the hematopoietic cells. Dissociated BM cells were then washed with 1% fetal bovine serum (FBS) in PBS. Cells were stained with any of the following antibodies for 20 min on ice: anti-CD16/32 (Invitrogen), lineage antibody cocktail-v450 (BD), anti-Sca-1 (Invitrogen, D7), anti-CD45 (BD, 30-F11), anti-CD45.1 (BD, A20 or Invitrogen, A20), anti-CD45.2 (Invitrogen, 104), anti-CD48 (Invitrogen, HM48-1), anti-CD150 (Invitrogen, 9D1 or BioLegend, TC15-12F12.2), anti-c-Kit (BioLegend, ACK2 or Invitrogen, ACK2), anti-CD34 (Invitrogen, RAM34), anti-CD41 (BioLegend, MWReg30), and anti-CD105 (BioLegend, MJ7/18). Goat anti-LepR-biotin polyclonal antibody (R&D Systems), Live/Dead dye (Invitrogen), anti-CD45 (BD, 30-F11), anti-Ter119 (Invitrogen, Ter119), anti-CD31 (BioLegend, MEC 13.3), and streptavidin (BD) were used to analyze the MSCs. Cells were then fixed and permeabilized using Cytofix/Cytoperm (BD) for 20 min during intracellular staining. Next, the cells were washed with Perm/Wash buffer (BD), incubated with rabbit anti-SCF polyclonal antibody (LSbio), and stained with Alexa Fluor 488-conjugated donkey anti-rabbit IgG (Invitrogen). Flow cytometry data were collected using LSR II (BD Biosciences), and the files were analyzed using FlowJo software (Tree Star).

### Reconstitution of BM cells

Recipient (CD45.2^+^) mice were treated with 10 Gy of total-body irradiation (cesium source irradiator; Precision X-Ray, North Branford, CT). The next day, recipient Gpr81^+/−^ or Gpr81^−/−^ mice received BM cells (1 × 10^7^ cells) from donor WT mice (CD45.1^+^). Recipient mice were then analyzed 12 weeks after irradiation.

### Administration of probiotics, *L. plantarum*, and lactate

Age- and sex-matched mice were orally administered probiotic VSL#3 (2 × 10^9^ CFU, Sigma-Tau Pharmaceuticals) in 100 μL of PBS and drinking water containing dl-lactate (10 mM, Sigma-Aldrich) daily for 1 week. *L. plantarum* strains were grown at 37 °C in MRS broth (BD Difco), as previously described^33^. Both *L. plantarum* strains were kindly provided by Dr. Siqing Liu, National Center for Agricultural Utilization Research, US Department of Agriculture. GF mice were orally administered 2 × 10^9^ CFU of WT *L. plantarum* or *L. plantarum* Δ*ldhD-*Δ*ldhL* strains.

### Irradiation-induced injury model

Mice were irradiated with 6 Gy of total-body irradiation (cesium source irradiator; Precision X-Ray, North Branford, CT) and analyzed at 1–2 weeks postirradiation. During the busulfan-induced injury model, mice were injected intraperitoneally with busulfan (25 mg/kg, Sigma-Aldrich) and analyzed after 1 week.

### Histology

Dissected bones were fixed in 4% paraformaldehyde for 1 day and then decalcified for 1 week in 0.5 M EDTA refreshed daily. The fixed tissues were dehydrated through a chain of graded ethanol baths and then embedded in paraffin. The paraffin-embedded blocks were cut into 5-μm-thick sections and stained with hematoxylin and eosin (H&E).

### Immunohistochemistry analysis

Bone sections were permeabilized in PBS containing 0.5% Triton X-100 at RT for 20 min and blocked with 5% BSA in PBS at RT for 1 h. The sections were then stained with primary antibodies against DAPI (Invitrogen), anti-VE-cadherin (R&D, 162709), anti-CD31 (BD, MEC 13.3), anti-SDF-1α (R&D, 79018), anti-endomucin (Abcam, V.7C7.1), rabbit anti-SCF polyclonal (Abcam), and goat anti-LepR-biotin polyclonal (R&D Systems) overnight at 4 °C. Sections were then washed with PBS and incubated with Alexa Fluor 488-conjugated donkey anti-rat IgG (Invitrogen), Alexa Fluor 546-conjugated donkey anti-rabbit IgG (Invitrogen), DyLight 488-conjugated streptavidin (BioLegend), or DyLight 647-conjugated streptavidin (BioLegend) at RT for 1 h. Fluorescence images of all samples were captured on an LSM 710 confocal microscope (Carl Zeiss). To measure the number of SCF^+^ cells, we counted LepR^+^SCF^+^ cells attached to endothelial cells.

### Immunocytochemistry analysis

Enzymatically digested BM cells were seeded in eight-well Lab-Tek II Chamber slides (5 × 10^4^ cells/well, Sigma-Aldrich). Cells were cultured in α-MEM (Gibco) supplemented with 10% FBS and 1% penicillin/streptomycin (Gibco) for 3 days and then treated with sodium l-lactate (5 mM, Sigma-Aldrich), 3,5-DHBA (0.2 mM, Sigma-Aldrich) or 3-OBA (3 mM, Sigma-Aldrich). Plated MSCs were fixed with Cytofix/Cytoperm (BD) for 20 min at 4 °C and subsequently washed with Perm/Wash buffer (BD) during immunocytochemistry. After blocking with 5% BSA in PBS for 1 h, MSCs were stained with DAPI, goat anti-LepR-biotin polyclonal (R&D Systems), anti-endomucin (Abcam, V.7C7.1), anti-SDF-1α (R&D, 79018), and rabbit anti-SCF polyclonal (Abcam) antibodies overnight at 4 °C. After one wash with Perm/Wash buffer, cells were stained with DyLight^TM^ 488-conjugated streptavidin (BioLegend) or Alexa Fluor 546-conjugated donkey anti-rabbit IgG (Invitrogen) as secondary antibodies. All samples were viewed under a confocal laser scanning microscope (Carl Zeiss).

### Colony-forming unit (CFU) assay

BM cells were suspended in MethoCult media (StemCell) as per the manufacturer’s instructions. Cells were then dispensed into 24- or 48-well culture plates (Thermo Scientific) using a syringe with a 16-gauge blunt needle (StemCell) and incubated at 37 °C in a 5% CO_2_ incubator. CFU-E and CFU-GM were counted after 2 and 12 days of culture, respectively. During the CFU-F analysis, BM MSCs were seeded in a six-well culture plate (Thermo Scientific) (1 × 10^6^ cells/mL) and cultured with α-MEM (Gibco) supplemented with 10% FBS and 1% penicillin/streptomycin (Gibco) and 10 µM ROCK inhibitors (Sigma). Cells were then washed with PBS to prevent the spread of hematopoietic cells and cultured with fresh medium. Colonies were counted after 7 days of culture by staining with 1% crystal violet (Sigma).

### Complete blood count analysis

Blood cells were analyzed using the ADVIA 2120i Hematology System (Siemens). Peripheral blood obtained from retro-orbital plexus bleeding was placed immediately into a microtainer blood collection tube (BD) with K_2_ EDTA. Samples were then gently mixed and stored on ice until analysis.

### Real-time PCR

RNA was extracted using the RNeasy mini kit (Qiagen) from the total BM cells isolated from the femur and tibia. Thereafter, RNA was converted to cDNA using Superscript II reverse transcriptase and oligo (dT) primers (Thermo Fisher). The cDNA was then used as the template for real-time qPCR conducted using SYBR green chemistry (Affymetrix) on an ABI 7500 real-time qPCR system (Applied Biosystems). The primer sequences used for real-time qPCR were as follows: *Scf*, 5′-GCCAGAAACTAGATCCTTTA-3′ and 5′-CATAAATGGTTTTGTGA CACTGACTCTG-3′; *SDF-1α*, 5′-GAGCCAACGTCAAGCATCTG-3′ and 5′-CGGGTC AATGCACACTTGT-3′; and β-actin, 5′-TGGAATCCTGTGGCATCCATGAAAC-3′ and 5′-TAAAACGCAGCTCAGTAACAGTCCG-3′.

### ELISA

Whole BM cells were homogenized with Tris-EDTA buffer (10 mM Tris-HCl, 1 mM EDTA, 0.05% sodium azide, 1% Tween-80, and protease inhibitor cocktail, Roche) and centrifuged at 11,000 × *g* for 10 min. The supernatant was collected, and the SCF concentration was measured with an SCF ELISA Kit (Abcam) as per the manufacturer’s instructions. Absorbance was measured at 450 nm using a spectrophotometer microplate reader (Bio-Rad).

### Lactate concentration analysis

Serum was obtained from retro-orbital plexus bleeding. The lactate concentration in serum was measured using a lactate assay kit (Biovision) as per the manufacturer’s instructions. Absorbance was measured at 570 nm using a spectrophotometric microplate reader (Bio-Rad).

### Statistics

Statistical analyses were performed with Prism software (GraphPad), and two-tailed *t*-tests were performed for pairwise and two independent group comparisons. Data are presented as the mean ± SEM, and a *P* value of <0.05 was considered statistically significant. The exact value of *n*, representing the number of mice in the experiments depicted, is indicated in the figure legends. Any additional technical replicates are described within the “Materials and methods” and “Results” sections.

## Results

### The Gpr81 signal regulates the proliferation of HSCs

**Fig. 1:**
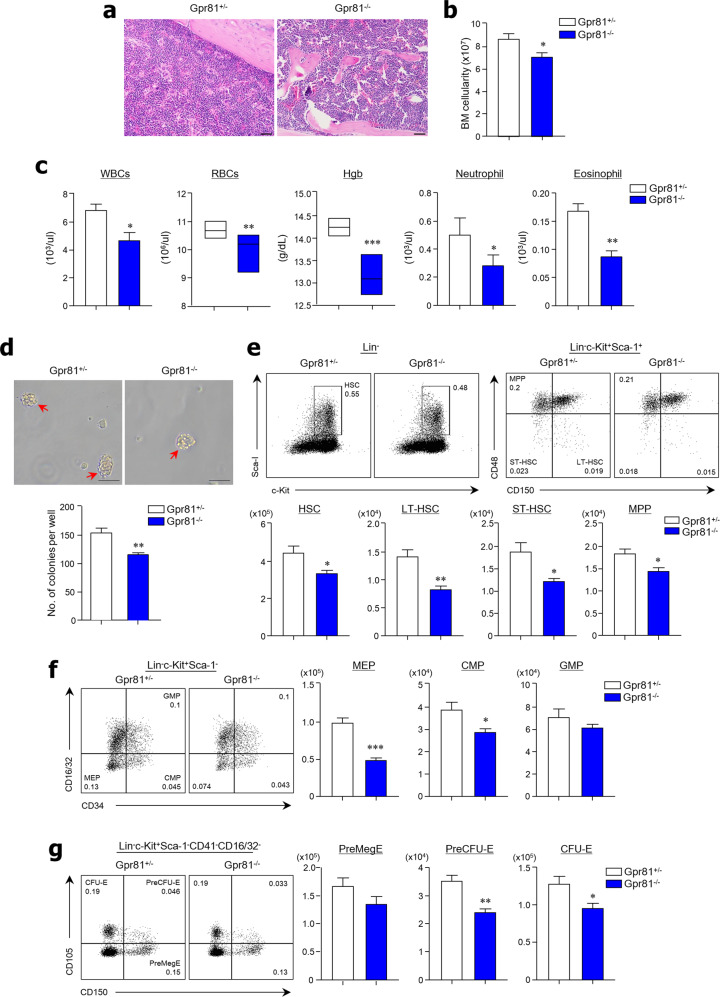
Gpr81-lactate signals play a crucial role in hematopoiesis and erythropoiesis. **a** Representative BM tissue sections stained with H&E. Scale bar = 100 μm. **b** BM cellularity from two tibias and femurs of Gpr81^+/−^ and Gpr81^−/−^ mice. **c** Complete blood cell count (CBC) analysis of WBCs, RBCs, hemoglobin (Hgb), neutrophils, and eosinophils in the peripheral blood of Gpr81^+/−^ and Gpr81^−/−^ mice. **d** Colony-forming unit-erythroid (CFU-E) assay using flushed total BM cells of Gpr81^+/−^ and Gpr81^−/−^ mice. Red arrows indicate colonies of CFU-E. Scale bar = 20 μm. **e** Representative FACS plots for total proportions (%) and absolute cell numbers of HSCs (c-Kit^+^Sca-1^+^lineage^−^; KSL), LT-HSCs (CD150^+^ CD48^−^ KSL), ST-HSCs (CD150^−^CD48^−^ KSL), and MPPs (CD150^−^CD48^+^ KSL) in total BM cells from two mouse tibias and femurs. CD150 (clone TC15-12F12.2) antibodies were used for analysis. **f** Representative FACS plots for total proportions (%) and absolute cell numbers of MEPs (CD16/32^−^CD34^−^c-Kit^+^Sca-1^−^Lin^−^), CMPs (CD16/32^−^CD34^+^ c-Kit^+^Sca-1^−^Lin^−^), and GMPs (CD16/32^+^CD34^+^ c-Kit^+^Sca-1^−^Lin^−^) in total BM cells. **g** Representative FACS plots for total proportions (%) and absolute cell numbers of PreMegEs (CD105^−^CD150^+^), PreCFU-Es (CD105^+^ CD150^+^), and CFU-Es (CD105^+^ CD150^−^) in total BM cells. CD150 (clone TC15-12F12.2) antibody was used for analysis. Data are shown as the mean ± SEM; comparisons were made using a two-tailed *t*-test, *n* = 3–5. *P < 0.05, **P < 0.01, ***P < 0.001. Data were combined from ≥3 independent experiments.

To determine the function of Gpr81 signaling on the proliferation of HSCs, we first analyzed the BM tissues of Gpr81^−/−^ mice using H&E staining. We observed a lower density and cellularity of BM cells in Gpr81^−/−^ mice than in Gpr81^+/−^ mice (Fig. 1a). We used Gpr81^+/−^ mice as controls because there were no differences in BM cellularity or HSC subsets compared with wild-type (WT) mice (Supplementary Fig. 1a, b). We obtained cells from two mouse femurs and tibias to measure the total number of BM cells. Significantly fewer cells were recovered from Gpr81^−/−^ mice than from Gpr81^+^^/−^ mice (Fig. 1b). In the peripheral blood, white blood cells, red blood cells (RBCs), and hemoglobin concentrations decreased significantly in the absence of Gpr81 signals. Additionally, neutrophil and eosinophil numbers decreased significantly in the absence of Gpr81 signals (Fig. 1c and Supplementary Fig. 1c). Next, we performed a CFU-erythroid (CFU-E) assay in vitro to investigate differentiation into erythroid progenitors. We found that Gpr81 deletion reduced the rate of HSC differentiation into erythroid progenitors (Fig. 1d). Flow cytometry was used to analyze HSC subsets using various differentiation markers [i.e., c-Kit(K)^+^ Sca-1(S)^+^ lineage (Lin)^−^ (KSL) HSCs, KSL^gated^CD150^+^CD48^−^ long-term (LT)-HSCs, KSL^gated^CD150^-^CD48^−^ short-term (ST)-HSCs, and KSL^gated^CD150^-^CD48^+^ multipotent progenitors (MPPs)]. Of note, there were significantly fewer HSCs, LT-HSCs, ST-HSCs, and MPPs found in the BM of Gpr81^−/−^ mice than in that of Gpr81^+/−^ mice (Fig. 1e). When we assessed all HSC subsets, we found that the absence of Gpr81 signals also caused a reduction in LT-HSCs and ST-HSCs (Supplementary Fig. 1d). These results indicate that Gpr81 signaling plays a crucial role in the self-renewal and maintenance of HSCs. We next analyzed the differentiation of myeloid and erythroid lineages in the BM of Gpr81^+/−^ and Gpr81^−/−^ mice. Among the myeloid lineages^34–36^, absolute cell numbers and total proportions of megakaryocyte-erythrocyte progenitors (MEPs; Lin^−^c-Kit^+^Sca-1^−^CD34^−^CD16/32^−^) were significantly lower in Gpr81^−/−^ mice than in Gpr81^+/−^ mice, whereas no differences were observed between granulocyte–macrophage progenitors (GMPs; Lin^−^c-Kit^+^Sca-1^−^CD34^+^ CD16/32^+^) (Fig. 1f). However, only absolute cell numbers were lower in the case of common myeloid progenitors (CMPs; Lin^−^c-Kit^+^Sca-1^−^CD34^+^ CD16/32^−^) in the BM of Gpr81^−/−^ mice than in the BM of Gpr81^+/−^ mice (Fig. 1f and Supplementary Fig. 1e). Erythropoiesis undergoes sequential differentiation steps: from pre-megakaryocyte-erythrocyte progenitors (PreMegEs; Lin^−^c-Kit^+^Sca-1^−^CD41^−^CD16/32^−^CD150^+^CD105^−^) to pre-CFU-erythrocytes (PreCFU-Es; Lin^−^c-Kit^+^Sca-1^−^CD41^−^CD16/32^−^CD150^+^ CD105^+^) and then to CFU-erythrocytes (CFU-Es; Lin^−^c-Kit^+^Sca-1^−^CD41^−^CD16/32^−^CD150^−^CD105^+^) and, finally, developed erythrocytes^34^. Consistent with the reduced numbers of RBCs in the peripheral blood, the BM of Gpr81^−/−^ mice possessed significantly reduced numbers of PreCFU-Es and CFU-Es compared with the BM of Gpr81^+/−^ mice (Fig. 1g and Supplementary Fig. 1f). These results suggest that Gpr81 signals play an indispensable role in BM cellularity.

### SCF from LepR^+^ MSCs in BM is regulated by the Gpr81 signal

**Fig. 2:**
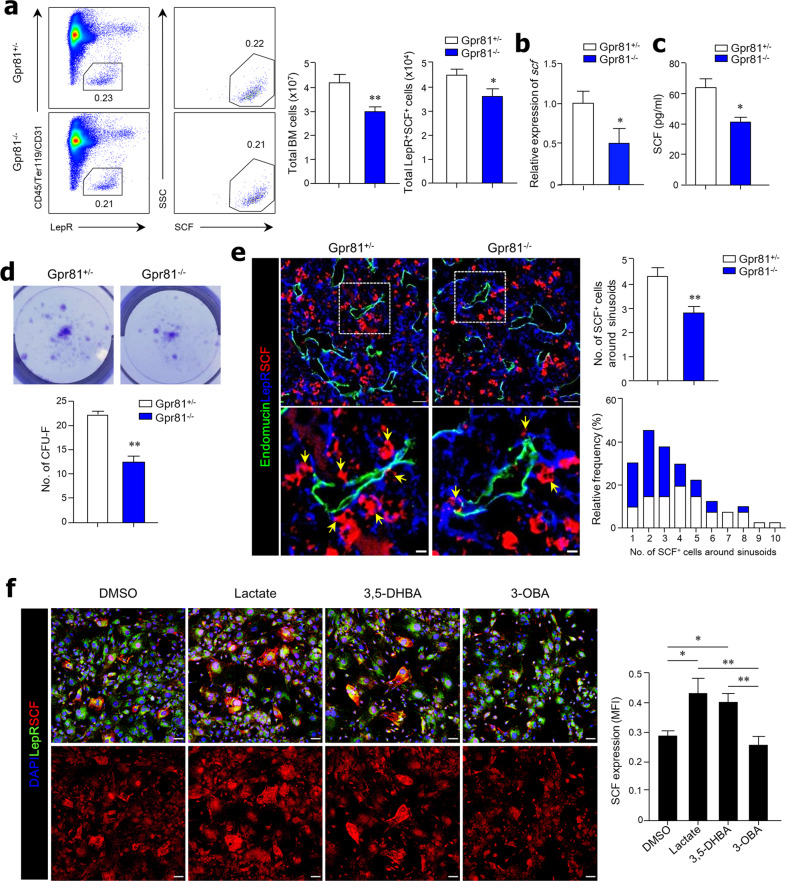
Lactate-Gpr81 signals regulate SCF production of LepR^+^ MSCs in BM. **a** Representative FACS plots for total proportions (%), recovered total BM cells, and absolute cell numbers of LepR^+^ SCF^+^ BM MSCs in total BM cells of Gpr81^+/−^ and Gpr81^−/−^ mice. **b** The gene expression level of *Scf* was measured by real-time PCR in total BM cells. Expression is relative to the β-actin gene. **c** The levels of SCF in BM homogenates were evaluated by ELISA. **d** Colony-forming unit fibroblast (CFU-F) assay using total BM cells obtained from Gpr81^+/−^ and Gpr81^−/−^ mice. Colonies were stained with crystal violet. **e** Immunohistochemical analysis of endomucin (green), LepR (blue), and SCF (red) expression in BM tissues and quantification of SCF^+^ cells around sinusoids of Gpr81^+/−^ and Gpr81^−/−^ mice. Scale bar = 20 μm (above) and 5 μm (below). Yellow arrows indicate LepR^+^SCF^+^ cells near the sinusoids. **f** Immunocytochemistry analysis of LepR (green) and SCF (red) expression in BM MSCs. Quantitation of SCF was measured by mean fluorescence intensity (MFI). Scale bar = 50 μm. Data are shown as the mean ± SEM; comparisons were made using the two-tailed *t*-test, *n* = 3–9. *P < 0.05, **P < 0.01. Data were combined from ≥2 independent experiments.

Given the critical role of LepR^+^ BM MSCs in the control of hematopoiesis through secretion of SCF and SDF-1α, we determined the involvement of LepR^+^ MSCs in reduced hematopoiesis in the absence of Gpr81. First, we confirmed that Gpr81 expression occurred in LepR^+^ MSCs located around the endomucin^+^ sinusoids of the BM in Gpr81^+/−^ mice, while no Gpr81 expression was detected in Gpr81^−/−^ mice (Supplementary Fig. 2a). BM MSCs were isolated, and SCF-secreting cells were analyzed by FACS to characterize LepR^+^ MSCs. While the numbers of LepR^+^ MSCs did not significantly differ between Gpr81^−/−^ mice and Gpr81^+/−^ mice, the numbers of recovered MSCs and SCF-secreting LepR^+^ MSCs were significantly reduced in the BM of Gpr81^−/−^ mice compared with Gpr81^+/−^ mice (Fig. 2a). In addition, the mRNA (Fig. 2b) and protein (Fig. 2c) levels of SCF in the homogenates of BM tissues were lower in Gpr81^−/−^ mice than in Gpr81^+/−^ mice. However, no significant differences in *Sdf-1α* mRNA expression were found in the BM tissues of Gpr81^+/−^ and Gpr81^−/−^ mice (Supplementary Fig. 2b). Likewise, the expression of SDF-1*α* from BM MSCs in vitro was unchanged in the presence of lactate or 3,5-DHBA (i.e., agonists of Gpr81) compared with DMSO alone or 3-OBA (i.e., antagonists of Gpr81) (Supplementary Fig. 2c). We next performed a CFU-fibroblast (CFU-F) assay to identify BM MSCs with spindle-like morphology, which have nonhematopoietic potential. The colony numbers of CFU-F significantly decreased in Gpr81^−/−^ mice compared with Gpr81^+/−^ mice (Fig. 2d). To confirm the secretion levels of SCF from LepR^+^ MSCs in BM tissues of Gpr81^−/−^ mice, we performed histological analysis by fluorescence staining using an endomucin antibody, a marker for endothelial cells. We found that the expression levels of SCF were significantly lower in BM tissues of Gpr81^−/−^ mice than in BM tissues of Gpr81^+/−^ mice (Fig. 2e). When SCF-expressing cells per endomucin^+^ sinusoid were counted, fewer SCF-expressing cells were identified in Gpr81^−/−^ mice than in Gpr81^+/−^ mice (Fig. 2e). In addition, SCF expression from BM MSCs in vitro increased in the presence of lactate or 3,5-DHBA compared with DMSO alone or 3-OBA (Fig. 2f). We therefore treated BM MSCs isolated from Gpr81^+/−^ and Gpr81^−/−^ mice to determine whether the increase in SCF expression occurs in a lactate-Gpr81-dependent manner. We also observed decreased SCF expression in Gpr81^−/−^ mouse-derived BM MSCs compared with Gpr81^+/−^ mouse-derived BM MSCs (Supplementary Fig. 2d). These results indicate that Gpr81 signals directly regulate SCF expression from LepR^+^ MSCs around BM sinusoids, which may control hematopoiesis.

### Hematopoietic recovery after hematologic stress requires Gpr81 signaling

**Fig. 3:**
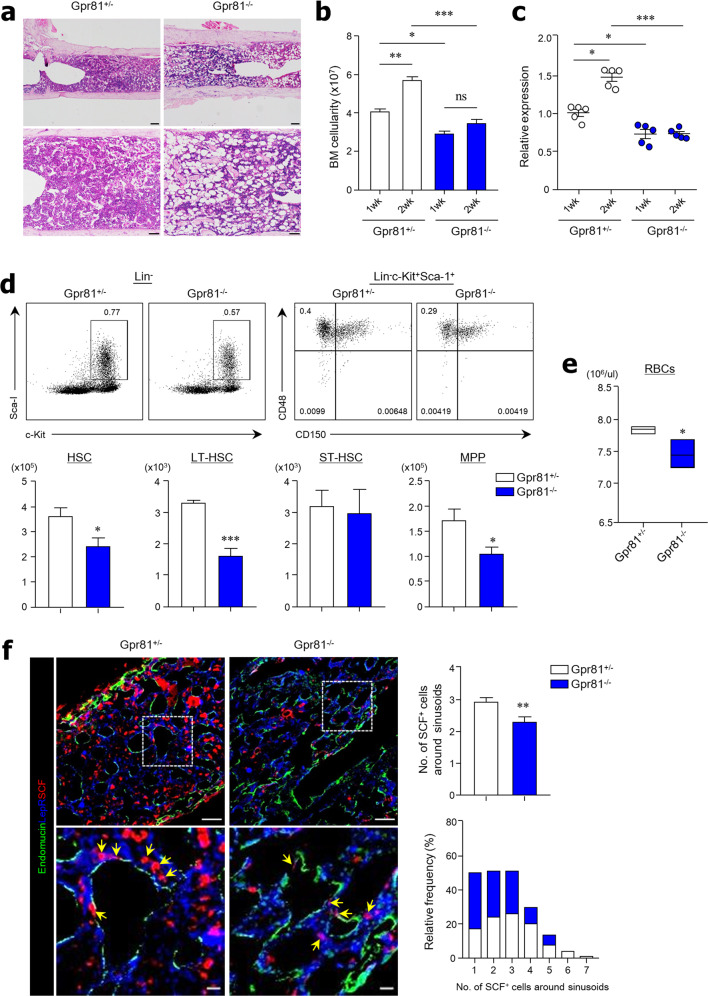
Lactate-Gpr81 signals affect the reconstitution of BM after 6 Gy irradiation. **a** H&E staining of BM tissue 2 weeks after irradiation. Scale bar = 200 μm (above) and 100 μm (below). **b** BM cellularity from two femurs and tibias of Gpr81^+/−^ and Gpr81^−/−^ mice after irradiation. **c** Expression levels of *Scf* mRNA in total BM cells were measured by real-time PCR after irradiation. The results shown are relative to β-actin gene expression. **d** Representative FACS plots for total proportions (%) and absolute cell numbers of HSCs, LT-HSCs, ST-HSCs, and MPPs in total BM cells 1 week after irradiation. CD150 antibodies (clone TC15-12F12.2) were used for the analysis. **e** RBC numbers in peripheral blood were evaluated 1 week after irradiation. **f** Immunohistochemical analysis of endomucin (green), LepR (blue), and SCF (red) expression in BM tissues 1 week after irradiation and quantification of SCF^+^ cells around sinusoids. Scale bar = 50 μm (above) and 10 μm (below). Data are shown as the mean ± SEM; comparisons were made using the two-tailed *t*-test, *n* = 3–6. *P < 0.05, **P < 0.01, ***P < 0.001. Data were combined from two independent experiments.

We next determined the hematopoietic recovery function of Gpr81 signaling by inducing hematologic stress with irradiation, chemotherapy (i.e., busulfan), or both, as described previously^23^. As anticipated, poor hematopoietic recovery was detected in the BM tissues of Gpr81^−/−^ mice compared with those of Gpr81^+/−^ mice 2 weeks after irradiation damage (Fig. 3a). Although total BM cells generally recovered in Gpr81^+/−^ mice by this time point, we observed delayed recovery in Gpr81^−/−^ mice (Fig. 3b). Notably, the expression levels of SCF were increased in the BM tissues of Gpr81^+/−^ mice at 2 weeks after damage, whereas less SCF expression was maintained in the BM tissues of Gpr81^−/−^ mice (Fig. 3c). Defects in hematopoietic recovery of Gpr81^−/−^ mice were also observed during FACS analysis (Fig. 3d). Absolute numbers of HSCs, LT-HSCs, and MPPs were significantly lower in the BM of Gpr81^−/−^ mice than in that of Gpr81^+/−^ mice 1 week after sublethal 6 Gy irradiation (Fig. 3d). However, the total proportions (%) of LT-HSCs but not other subsets were significantly reduced in the absence of Gpr81 signaling (Supplementary Fig. 3a). Furthermore, absolute numbers of BM subsets of myeloid (i.e., MEPs, CMPs, and GMPs) and erythroid (i.e., PreMegEs, PreCFU-Es, and CFU-Es) lineages did not recover in Gpr81^−/−^ mice compared with those of Gpr81^+/−^ mice (Supplementary Fig. 3b, c). Lower numbers of recovered RBCs in the peripheral blood were also noted in Gpr81^−/−^ mice than in Gpr81^+/−^ mice (Fig. 3e). Importantly, SCF expression on LepR^+^ MSCs around endomucin^+^ sinusoids was much higher in the BM tissues of Gpr81^+/−^ mice than in those of Gpr81^−/−^ mice (Fig. 3f). We obtained similar results when the BM was subjected to chemotherapy (i.e., busulfan). Hematopoietic recovery was reduced in BM tissues of Gpr81^−/−^ mice compared with those of Gpr81^+/−^ mice 1 week after busulfan injection (Supplementary Fig. 4a). To determine the differentiation of myeloid and erythroid lineages in the BM of Gpr81^+/−^ and Gpr81^−/−^ mice, we assessed CFU-granulocyte–macrophage progenitors (CFU-GM) and colony-forming unit-erythroids (CFU-E) after busulfan injection. As expected, depletion of Gpr81 impaired HSC differentiation into granulocyte–macrophage progenitors (Supplementary Fig. 4b) and erythroid progenitors (Supplementary Fig. 4c) after busulfan. Consistent with the irradiation injury results, SCF expression on LepR^+^ MSCs around endomucin^+^ sinusoids was significantly reduced in the BM tissues of Gpr81^−/−^ mice compared with those of Gpr81^+/−^ mice (Supplementary Fig. 4d). We also measured the survival rates of Gpr81^+/−^ and Gpr81^−/−^ mice after lethal 10 Gy irradiation, but no differences in hematopoietic recovery function were observed (data not shown). Overall, the data suggest that Gpr81 signals control SCF expression in LepR^+^ MSCs in BM tissues and subsequently play an indispensable role in BM reconstitution.

### Gpr81 signaling regulates HSCs

**Fig. 4:**
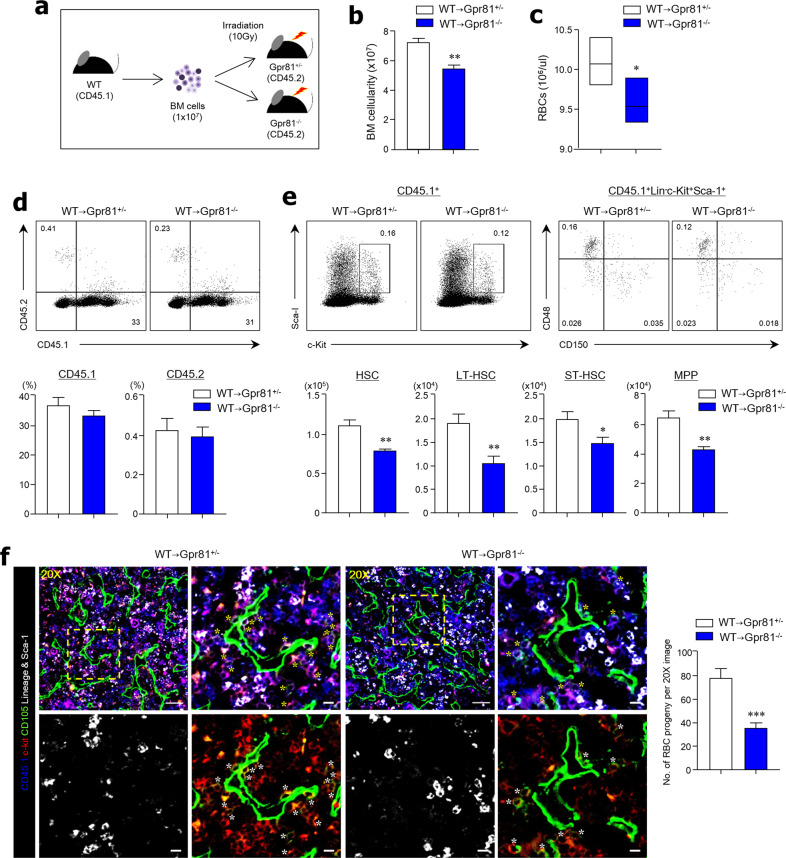
The BM environment accelerates the proliferation of HSCs in a Gpr81-dependent manner. **a** Scheme for BM reconstitution assay model. Ten Gy-irradiated Gpr81^+/−^ and Gpr81^−/−^ mice were transplanted with BM cells from donor CD45.1^+^ WT mice. Recipient Gpr81^+/−^ and Gpr81^−/−^ mice were analyzed 3 months after BMT. **b** BM cellularity from two mouse femurs and tibias. **c** RBCs in peripheral blood of recipient mice. **d** Representative FACS plots and total proportions (%) of CD45.1 and CD45.2 in BM cells of recipient mice. **e** Representative FACS plots for total proportions (%) and absolute cell numbers of HSCs, ST-HSCs, LT-HSCs, and MPPs in the BM cells of recipient mice. CD150 (clone TC15-12F12.2) antibody was used for analysis. **f** Immunohistochemical analysis of CD45.1 (blue), c-Kit (red), CD105 (green), Lineage and Sca-1 (white) expression in BM tissues. The numbers of RBC progeny (CD45.1^+^ Lineage^−^ Sca-1^−^ c-Kit^+^ CD105^+^) were measured in images at ×20 magnification. Scale bar = 50 μm (×20 image) and =10 μm (cropped image). Yellow and white asterisks indicate RBC progeny (CD45.1^+^Lingeage^−^Sca-1^−^c-Kit^+^CD150^+^). Data are shown as the mean ± SEM; comparisons were made using the two-tailed *t*-test, *n* = 5–6. *P < 0.05, **P < 0.01, ***P < 0.001. Data were repeated twice in independent experiments.

Irradiation-resistant CD45.1^+^ WT BM cells were then transferred into lethally irradiated (10 Gy) CD45.2-expressing Gpr81^+/−^ and Gpr81^−/−^ mice (Fig. 4a). As predicted, BM cellularity and peripheral RBC numbers were diminished in Gpr81^−/−^ mice compared with Gpr81^+/−^ mice 12 weeks after adoptive transplantation (Figs. 4b, c). We then confirmed that donor-derived CD45.1^+^ cells were chimerized to approximately 35% in recipient mice, while recipient-derived CD45.2^+^ cells had almost disappeared (Fig. 4d). Of note, the reconstitution capacity of CD45.1^+^ HSCs was significantly reduced in the BM of Gpr81^−/−^ mice compared with Gpr81^+/−^ mice. Furthermore, absolute cell numbers of HSC subsets (i.e., LT-HSCs, ST-HSCs, and MPPs) were significantly lower in the BM of Gpr81^−/−^ mice than in Gpr81^+/−^ mice (Fig. 4e and Supplementary Fig. 5a). In addition, myeloid progenitor subsets (i.e., MEPs, CMPs, and GMPs) and PreMegEs were lower in the BM of Gpr81^−/−^ mice than in Gpr81^+/−^ mice (Supplementary Fig. 5b, c). We further evaluated the differentiation function of donor-derived CD45.1 cells into RBC progeny. The proportions of RBC progenitors (i.e., CD45.1^+^ lineage^−^ Sca-1^−^cKit^+^ CD105^+^) were significantly lower in the BM tissue of Gpr81^−/−^ mice than in that of Gpr81^+/−^ mice (Fig. 4f). Therefore, our data indicate that the self-renewal and differentiation functions of HSCs are supported by Gpr81 signaling.

### Oral feeding with lactate-producing bacteria (LAB) accelerates hematopoiesis and erythropoiesis in the BM

**Fig. 5:**
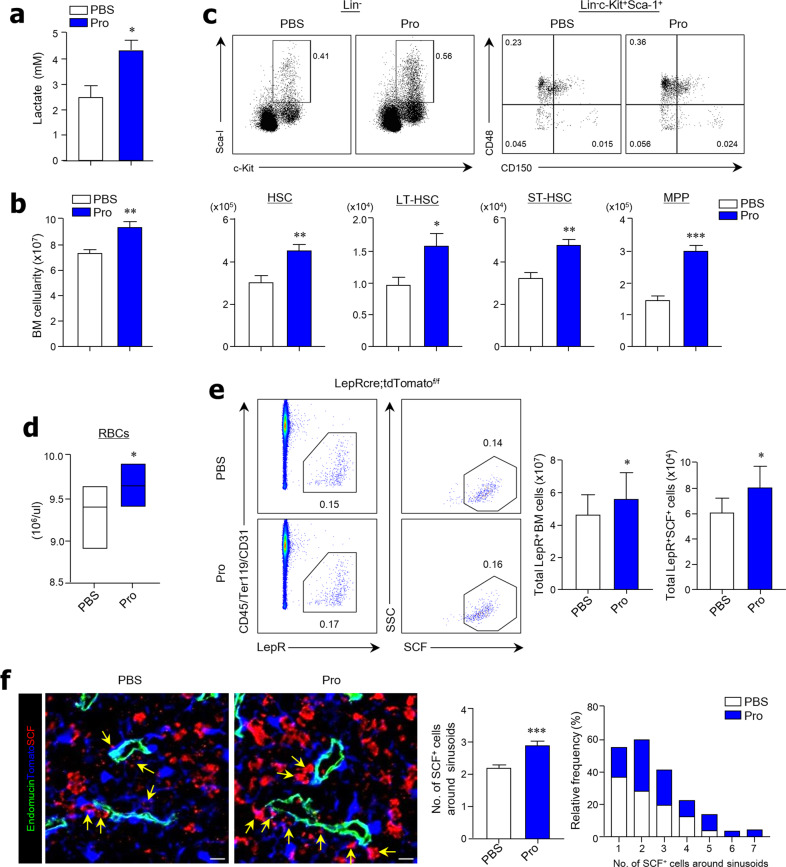
LAB-rich probiotics enhance LepR^+^ BM MSCs and the proliferation of HSCs. **a** Measurement of lactate in serum 1 week after administration of VSL#3. **b** BM cellularity from two femurs and tibias of C57BL/6 mice fed VSL#3 or vehicle for 1 week. **c** Representative FACS plots for total proportions (%) and absolute cell numbers of HSCs, LT-HSCs, ST-HSCs, and MPPs in total BM cells of C57BL/6 mice. **d** Measurement of RBC numbers in the peripheral blood of C57BL/6 mice. **e** Representative FACS plots for total proportions (%), recovered total BM cells, and absolute cell numbers of LepR^+^SCF^+^ BM MSCs in total BM cells obtained from LepR-cre;tdTomato mice. **f** Immunohistochemical analysis of endomucin (green), LepR (blue), and SCF (red) expression in BM tissues and quantification of SCF^+^ cells around sinusoids of LepR-cre;tdTomato mice. Scale bar = 10 μm. Yellow arrows indicate LepR^+^SCF^+^ cells near the sinusoids. Data are shown as the mean ± SEM; comparisons were made using the two-tailed *t*-test, *n* = 5–7. *P < 0.05, **P < 0.01, ***P < 0.001. Data were combined from ≥4 independent experiments.

To identify the role of LAB-type symbionts in hematopoiesis and erythropoiesis, we orally administered human-use probiotics (VSL#3) containing LAB (e.g., *Bifidobacterium* and *Lactobacillus* spp.) to WT mice daily for 1 week. Probiotic administration increased lactate concentrations in serum (Fig. 5a), and total numbers of BM cells increased significantly in mice receiving probiotics compared with mice receiving vehicle alone (Fig. 5b). In addition, significantly higher numbers and proportions (%) of HSCs, LT-HSCs, and ST-HSCs were seen in the BM of mice receiving LAB-type probiotics than in mice receiving vehicle alone (Fig. 5c and Supplementary Fig. 6a). Furthermore, probiotic administration appeared to increase the differentiation patterns of myeloid lineages (Supplementary Fig. 6b), accelerate erythropoiesis (i.e., PreMegEs, PreCFU-Es, and CFU-Es) (Supplementary Fig. 6c), and increase peripheral RBC numbers (Fig. 5d). We next further investigated the activation of SCF expression from LepR^+^ MSCs using LepRcre;tdTomato mice. Of note, oral administration of LAB-type probiotics accelerated the numbers of LepR^+^ MSCs and SCF expression by LepR^+^ MSCs in the BM (Fig. 5e). Furthermore, SCF secretion from LepR^+^ MSCs around endomucin^+^ sinusoids increased in the BM tissues in mice receiving LAB-type probiotics compared with mice receiving vehicle alone (Fig. 5f). These results suggest that microbiota-derived lactate may be associated with self-renewal and proliferation of BM cells through SCF secretion by LepR^+^ MSCs in a Gpr81-dependent manner.

### Oral feeding with lactate enhances hematopoiesis and erythropoiesis in BM

**Fig. 6:**
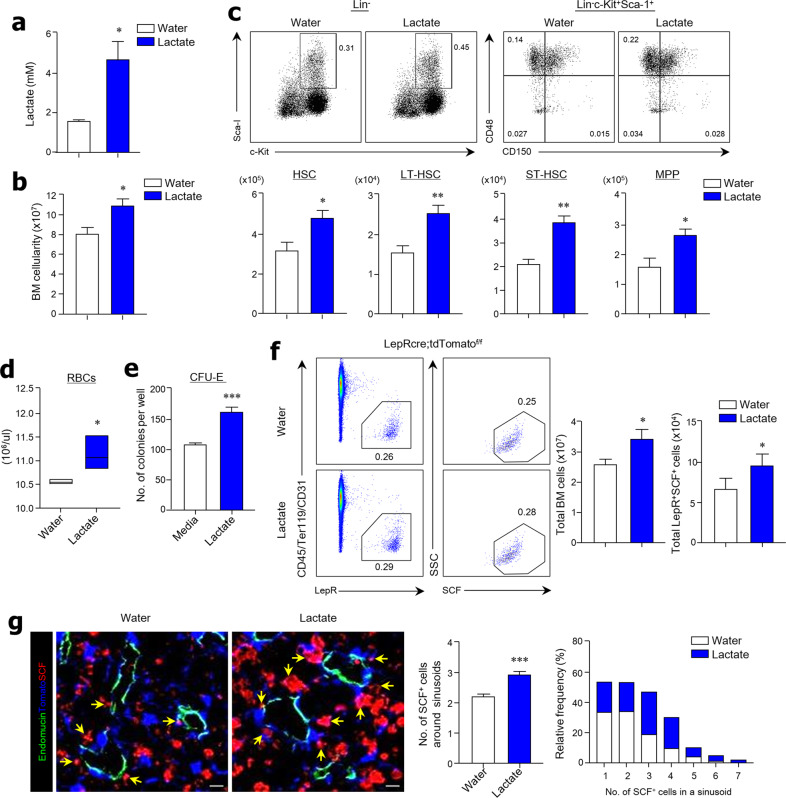
Lactate administration promotes SCF expression in LepR^+^ MSCs and the proliferation of HSCs. **a** Measurement of lactate in serum on day 1 after lactate feeding. **b** BM cellularity from two femurs and two tibias of C57BL/6 mice that were given drinking water supplemented with lactate (10 mM) for 1 week. **c** Representative FACS plots for total proportions (%) and absolute cell numbers of HSCs, LT-HSCs, ST-HSCs, and MPPs in total BM cells. **d** RBC numbers in the peripheral blood of C57BL/6 mice. **e** Colony-forming unit-erythroid (CFU-E) assay using flushed total BM cells of C57BL/6 mice in the presence of lactate (10 mM) in vitro. **f** Representative FACS plots for total proportions (%), recovered total BM cells, and absolute cell numbers of LepR^+^SCF^+^ BM MSCs in total BM cells from LepR-cre;tdTomato mice. **g** Immunohistochemical analysis of endomucin (green), LepR (blue), and SCF (red) expression in BM tissues and quantification of SCF^+^ cells around sinusoids of LepR-cre;tdTomato mice. Yellow arrows indicate LepR^+^SCF^+^ cells near sinusoids. Scale bar = 10 μm. Data are shown as the mean ± SEM; comparisons were made using the two-tailed *t*-test, *n* = 3–5. *P < 0.05, **P < 0.01, ***P < 0.001. Data were repeated twice in independent experiments.

To further clarify the effects of gut microbiota-derived lactate on HSC proliferation, WT mice were provided drinking water ad libitum supplemented with 10 mM lactate for 1 week. We confirmed that the lactate concentration in serum increased after lactate feeding (Fig. 6a). The total number of BM cells was significantly augmented in mice given lactate compared with mice given drinking water alone (Fig. 6b). As expected, the absolute numbers and proportions (%) of HSCs, LT-HSCs, ST-HSCs, and MPPs increased considerably in the BM due to lactate feeding (Fig. 6c and Supplementary Fig. 7a). There was also a significant increase in absolute cell numbers of myeloid lineage progenitors (i.e., MEPs, CMPs, and GMPs) and erythroid lineage progenitors (i.e., PreMegEs, PreCFU-Es, and CFU-Es) (Supplementary Fig. 7b, c). To support the acceleration of erythropoiesis, we found more RBCs in the peripheral blood of lactate-fed mice than in water-fed mice (Fig. 6d). In addition, the in vitro CFU-E assay indicated that lactate feeding increased HSC differentiation into erythroid progenitors (Fig. 6e). Furthermore, we found that lactate feeding enhanced the numbers of LepR^+^ MSCs expressing SCF in the BM (Fig. 6f). Likewise, lactate-fed mice had higher numbers of SCF-secreting LepR^+^ MSCs around endomucin^+^ sinusoids in the BM tissues than water-fed control mice (Fig. 6g). These results suggest that symbiont-derived lactate might play an indispensable role in hematopoiesis and erythropoiesis in the BM through the control of SCF secretion.

### LAB feeding results in self-renewal of HSCs in GF mice

**Fig. 7:**
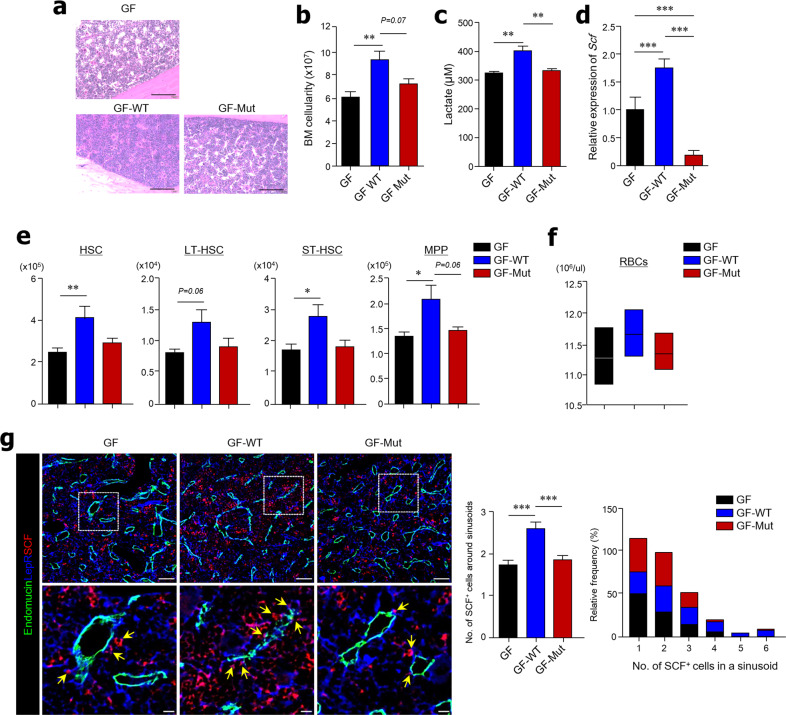
Oral administration of *Lactobacillus plantarum* promotes self-renewal of HSCs in GF mice. Wild-type (WT) *L. plantarum* or *L. plantarum* Δ*ldhD-*Δ*ldhL* mutant (Mut) strains were orally administered to GF mice once and analyzed 16 weeks later. **a** H&E-stained BM tissues. Scale bar = 100 μm. **b** BM cellularity of two mouse femurs and tibias. **c** Measurement of lactate in serum. **d** Expression levels of *SCF* mRNA in total BM cells. **e** Absolute numbers of HSCs, LT-HSCs, ST-HSCs, and MPPs in total BM cells. CD150 (clone TC15-12F12.2) antibody was used for FACS analysis. **f** RBCs measured in peripheral blood. **g** Immunohistochemical analysis of endomucin (green), LepR (blue), and SCF (red) expression in BM tissues. Scale bar = 50 μm (above) and 10 μm (below). Yellow arrows indicate LepR^+^SCF^+^ cells located near sinusoids. Data are shown as the mean ± SEM; comparisons were made by two-tailed *t*-tests, *n* = 3–5. *P < 0.05, **P < 0.01, ***P < 0.001. Data were combined from two independent experiments.

To directly confirm that microbiota-derived lactate plays a role in the BM niche, we fed GF mice once with WT *Lactobacillus plantarum* and *L. plantarum* strains lacking lactate dehydrogenase activity (*L. plantarum ΔldD-ΔldhL*) and analyzed the effects after 16 weeks. First, we confirmed that both strains were well colonized in GF mice (Supplementary Fig. 8a). Oral administration of WT *L. plantarum* resulted in a higher BM density in GF mice than in naïve and mutant *L. plantarum*-fed GF mice (Fig. 7a). Additionally, total BM cell numbers significantly increased in WT *L. plantarum*-fed GF mice compared with naïve and mutant *L. plantarum*-fed GF mice (Fig. 7b). We measured the serum lactate levels of GF mice after oral feeding with WT *L. plantarum* to confirm circulation of lactate from the gut into the BM. The serum of WT *L. plantarum*-fed GF mice had higher lactate levels than that of naïve and mutant-fed GF mice (Fig. 7c). In addition, *Scf* mRNA levels in the BM cells of GF mice significantly increased after a single administration of WT *L. plantarum* (Fig. 7d). Importantly, significant expansion of HSCs, LT-HSCs, ST-HSCs, and MPPs was observed in the BM of GF mice 16 weeks after a single administration of WT *L. plantarum* (Fig. 7e). Moreover, myeloid lineage progenitors (i.e., MEPs, CMPs, and GMPs) and RBC lineage progenitors (i.e., PreMegEs, PreCFU-Es, and CFU-Es) also increased significantly compared with normal GF mice and Mut-fed GF mice (Supplementary Fig. 8b, c). BM cell suspensions from WT *L. plantarum*-fed GF mice also had a marked red appearance compared with naïve and mutant *L. plantarum*-fed GF mice (Supplementary Fig. 8d). Subsequently, we observed increased numbers of RBCs in the peripheral blood of WT *L. plantarum*-fed GF mice (Fig. 7f). Importantly, we observed greater expression of SCF on LepR^+^ MSCs around the endomucin^+^ sinusoids of BM tissue in GF mice 16 weeks after a single administration of WT *L. plantarum* compared with naïve and mutant strain-fed GF mice (Fig. 7g). Therefore, these results indicate that gut microbiota-derived lactate is associated with SCF expression on LepR^+^ MSCs, which subsequently controls hematopoiesis and erythropoiesis in the BM.

## Discussion

Our study demonstrates that microbiota-derived lactate accelerated SCF secretion from LepR^+^ niche cells around endomucin^+^ sinusoids in BM tissue and that this process is associated with BM cellularity. Microbiota-derived lactate modulated not only hematopoiesis and erythropoiesis but also hematologic recovery after radiation and busulfan exposure. The interplay between microbiota-derived lactate and HSC proliferation appears to result from lactate-Gpr81 interactions. Single colonization with LAB in GF mice resulted in activation of SCF in LepR^+^ niche cells and HSC proliferation, further supporting the notion that microbiota-derived lactate has a role in regulating BM cellularity.

Three mechanisms have been proposed to explain the effect of gut microbial metabolites on host homeostasis in distant tissues beyond the gut^37^. First, some microbial metabolites could enter the systemic circulation and directly modulate immune cell function in distant target tissues. Research has demonstrated that gut microbiota-derived SCFAs modulate pancreatic endocrine cells to promote the synthesis of key anti-inflammatory molecules^5^. Second, microbial metabolites may locally educate immune cells in the gut, and these educated cells could then migrate to target tissues and impact disease processes. For instance, previous research has shown that the probiotic *Lactobacillus rhamnosus*-derived butyrate increases the frequency of intestinal Treg cells, which then migrate to the BM and cause BM CD8^+^ T cells to activate osteoblasts^38^. Last, microbiota-derived metabolites may enter the systemic circulation and indirectly affect distant target tissues, a concept supported by our findings that microbiota-derived lactate can modulate hematopoiesis in the BM.

Despite the anatomical separation of the gut and systemic compartments, experimental evidence suggests that various microbiota-derived metabolites present in the bloodstream contribute to steady-state hematopoiesis^9,13,39^. For instance, microbiota-derived SCFAs (mainly propionate) promote DC precursor activation in the BM and their release into the bloodstream, consequently seeding the lungs with DCs with dominant phagocytic capacity^4^. Moreover, in response to circulating bacteria-derived TLR ligands, which may originate from bacterial infections or the gut microbiota, BM MSCs and their progeny express MCP-1 and promote CCR2^+^ monocyte trafficking into the bloodstream^40^. Similarly, systemic recognition of microbiota-derived TLRs is necessary to maintain a sufficient pool of BM myeloid cell lineages^11^. Furthermore, BM MSCs of GF mice could sense NOD1 ligands after in vivo administration, leading to the expression of known hematopoietic factors, including IL-7, Flt3L, SCF, and thrombopoietin, which can then lead to the restoration of HSC numbers to levels seen in conventional mice^41^. Regarding the effect of hematopoiesis caused by lactate, an earlier study reported that administration of lactate promoted erythropoiesis in BM and increased hemoglobin expression in leukemic K562 cells in vitro^42^. Of note, lactate is released from human neutrophils, and increased levels are used as markers for detecting sepsis in humans^43^. A more recent study revealed that lactate released by inflammatory BM neutrophils induced their mobilization through endothelial lactate-Gpr81 signaling^44^. While systemic penetrance of microbial metabolites may result from active transport mechanisms or passive diffusion, we suggest that other potential metabolites (e.g., lactate) from gut microbiota that control hematopoiesis through activation of LepR^+^ niche cells in BM sinusoids are present.

BM HSCs either self-renew or differentiate into restricted progenitors in a process dependent upon SCF, SDF-1α, and pleiotrophin and are primarily produced by LepR^+^ MSCs and endothelial cells in perisinusoidal niches^24,25,45^. In contrast, MSCs of the endosteal niche that are distanced from the sinusoids mainly contribute to maintaining HSC quiescence^46,47^. Therefore, we assume that lactate in our study had a greater effect on perisinusoidal MSCs than MSCs located farther from the sinusoids. A recent study by Comazzetto et al.^31^ supports the concept that SCF produced by perisinusoidal LepR^+^ MSCs provides a suitable microenvironment in the BM to maintain c-kit^+^-restricted progenitors^31^. c-kit^+^-restricted progenitors and early erythroid progenitors primarily reside in perisinusoidal niches adjacent to LepR^+^ MSCs. Additionally, c-kit^+^-restricted hematopoietic progenitors (i.e., CMPs, CLPs, GMPs, MEPs, PreMegEs, and CFU-Es) and myeloid and erythroid blood cells were depleted when *Scf* was conditionally deleted in LepR^+^ MSCs^31^. Our data have shown that feeding LAB to GF mice causes activation of SCF secretion by LepR^+^ niche cells in BM sinusoids and the expansion of many types of HSC progenitors and erythroid blood cells. These data support the hypothesis that microbiota-derived lactate in systemic circulation stimulates adjacent SCF-secreting LepR^+^ niche cells in BM sinusoids.

Our previous study demonstrated that renewal and differentiation of intestinal stem cells were impaired in Gpr81^−/−^ mice due to reduced expression and secretion of Wnt3 from Paneth and stromal cells^23^. Similarly, we found that the functionality and proportion of HSCs were significantly lower in Gpr81^−/−^ mice. These mice also showed significantly reduced lineage differentiation in MEPs, which was found to be associated with erythropoiesis. We observed that CFU-Es, the precursor of RBC differentiation, and RBCs decreased in the absence of Gpr81 signaling. Therefore, our data show that lactate-Gpr81 signaling regulates myeloid lineages and is involved in the differentiation of erythroid lineages. Previous findings note that erythroid differentiation is related to SCF expression by LepR^+^ MSCs in BM sinusoids^31^.

Individuals with impaired hematopoiesis are susceptible to several diseases, including anemia and aging. Chronic anemia is caused by inhibition of erythropoiesis by chronic stimulation of inflammatory cytokines such as IFN-γ^48^. Previous studies reported that lactate-Gpr81 signals inhibit the proinflammatory response of macrophages^49^. Together with these studies, lactate-Gpr81 signal-induced hematopoiesis and erythropoiesis may be potential therapeutic strategies for patients with chronic anemia.

**Fig. 8:**
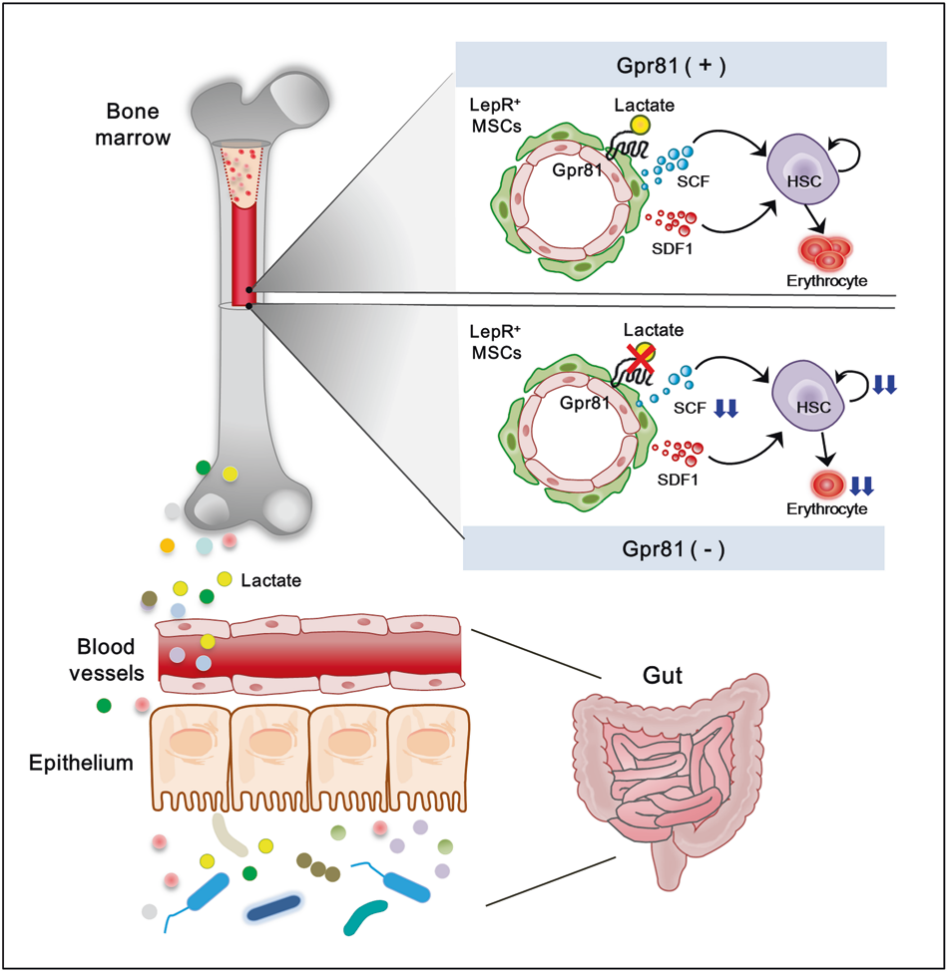
Overview of the mechanism by which microbiota-derived lactate regulates hematopoiesis and erythropoiesis. Gut microbial products such as lactate reach the bone marrow through the blood circulation and stimulate SCF secretion from LepR^+^ stromal cells around BM sinusoids in a Gpr81 signaling-dependent manner. Furthermore, this SCF promotes BM cellularity.

In conclusion, we identified the molecular mechanism by which microbiota-derived lactate activates SCF secretion from LepR^+^ cells in the BM and regulates hematopoiesis and erythropoiesis in a Gpr81-dependent manner (Fig. 8). These insights may open new avenues for affecting HSC differentiation in BM niches through microbiota modulation and represent potential new therapeutic uses of LAB in patients with anemia.

## Supplementary information

Supplementary Information
