# Supplementary material for: Microbiota-derived lactate promotes hematopoiesis and erythropoiesis by inducing stem cell factor production from leptin receptor+ niche cells
Source: Exp Mol Med. 2021 Sep 9;53(9):1319–31. doi: 10.1038/s12276-021-00667-y (PMC8492757; doi:10.1038/s12276-021-00667-y)
Supplement: Supplementary file 1 — Supplementary Information [file 12276_2021_667_MOESM1_ESM.pdf]

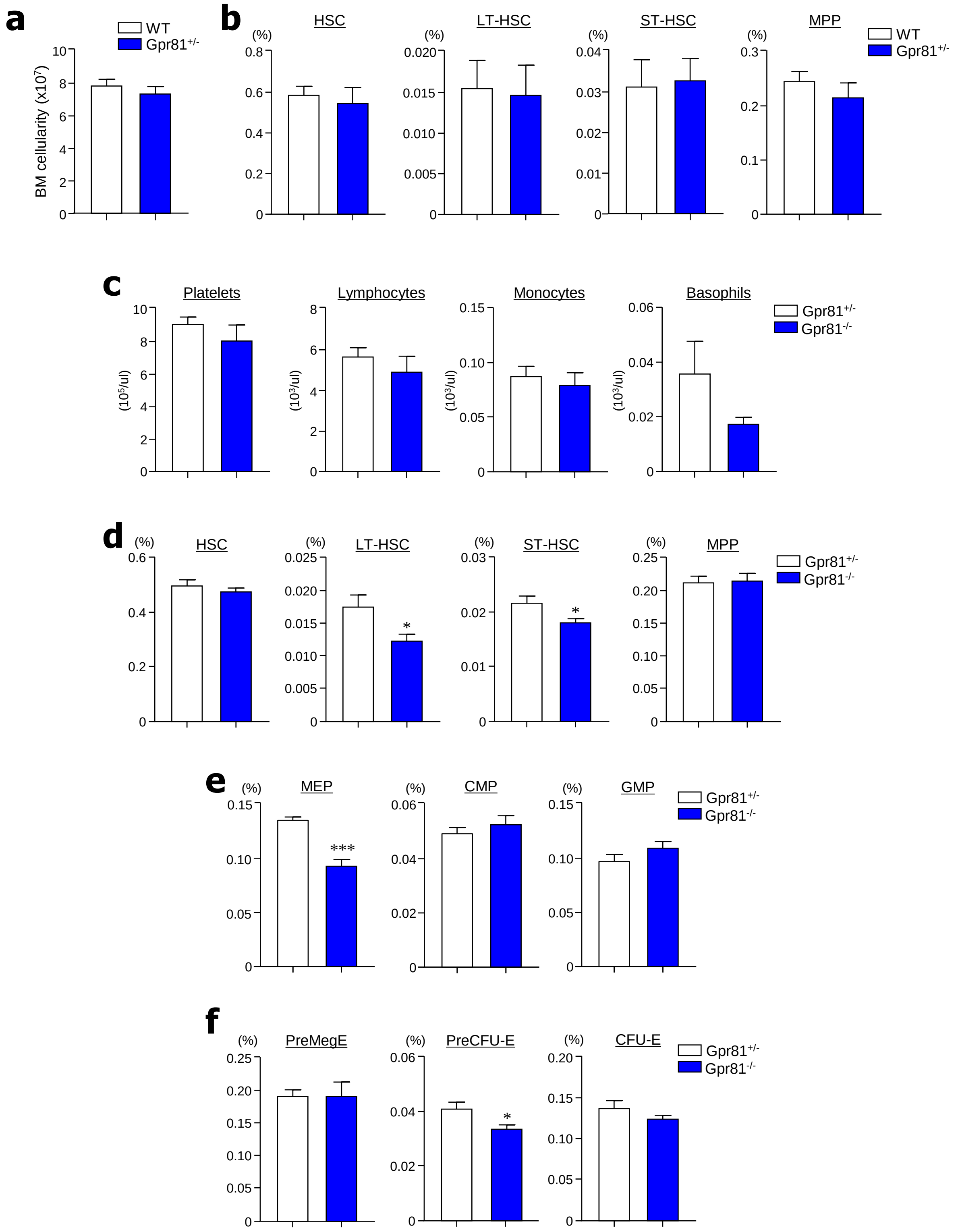

Supplementary Figure. 1 Lactate-Gpr81 signals regulate BM hematopoiesis. (**a**) BM cellularity from two tibiae and two femurs of each wild-type (WT) and Gpr81$^{+/-}$ mice. (**b**) Percentages of HSCs, LT-HSCs, ST-HSCs, and MPPs in total BM cells of WT and Gpr81$^{+/-}$ mice. (**c**) CBC analysis for numbers of platelets, lymphocytes, monocytes, and basophils in the peripheral blood. (**d**) Percentages of HSCs, LT-HSCs, ST-HSCs, and MPPs in total BM cells. (**e**) Percentages of MEPs, CMPs, and GMPs in total BM cells. (**f**) Percentages of PreMegEs, PreCFU-Es, and CFU-Es in total BM cells. CD150 antibody (clone TC15-12F12.2) was used for FACS analysis shown in B, D, and F. Data from >3 independent experiments are shown as mean ± SEM; comparisons were made by two-tailed *t*-test, n = 3-5. *$p < 0.05$, ***$p < 0.001$.

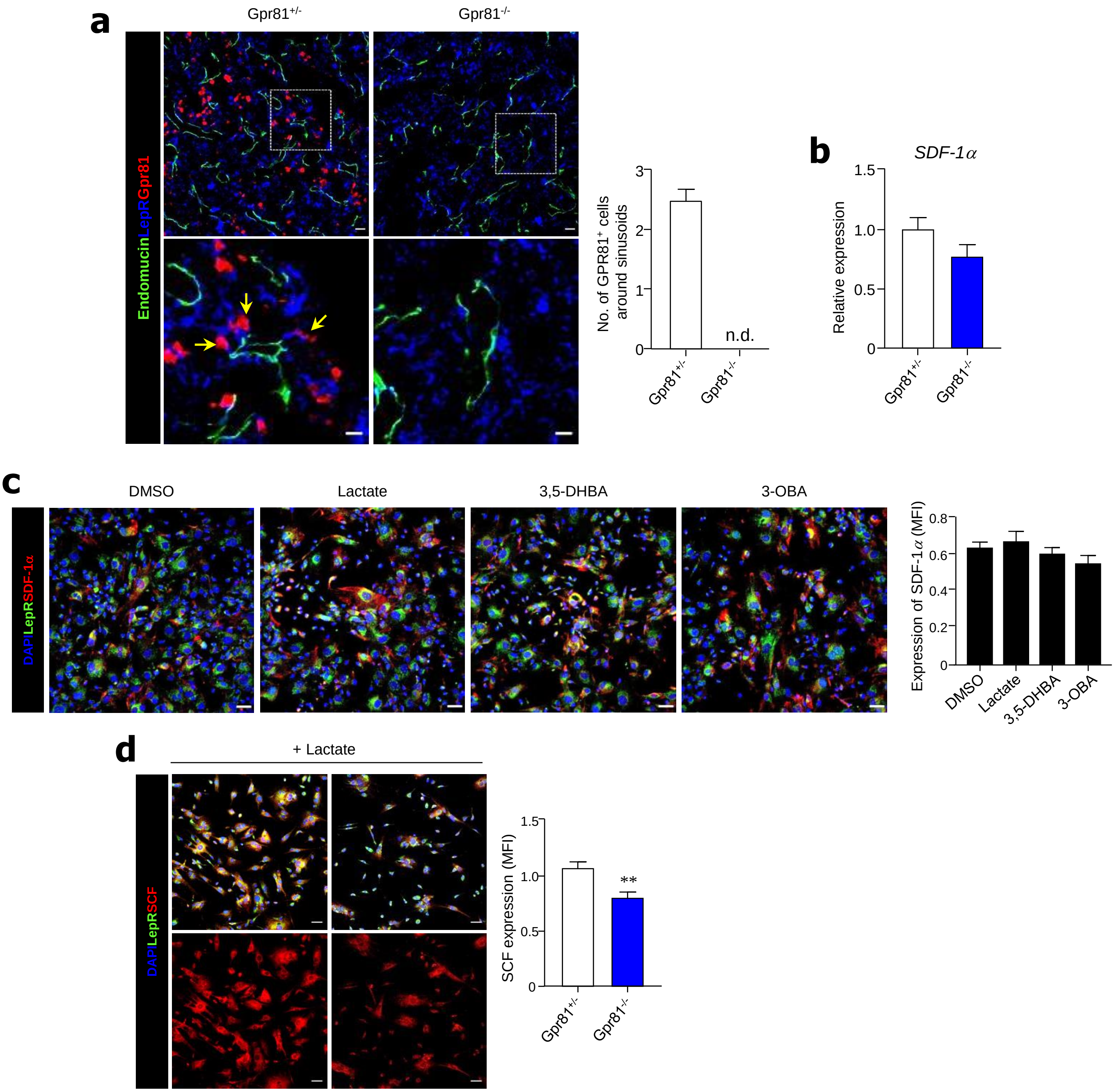

Supplementary Figure. 2 Expression levels of Gpr81 on BM MSCs. (**a**) Immunohistochemical analysis for endomucin (green), LepR (blue), and Gpr81 (red) expression in BM tissues. Scale bar = 20 µm (above) and = 10 µm (below). (**b**) Expression levels of *Sdf-1*a mRNA in total BM cells measured by real-time PCR. Results shown are relative to β-actin gene expression. (**c**) Immunocytochemistry analysis for DAPI (blue), LepR (green), and SDF-1a (red) expression from BM MSCs in the presence of lactate, 3,5-DHBA, and 3-OBA. (**d**) Immunocytochemistry analysis for DAPI (blue), LepR (green), and SCF (red) expression from BM MSCs in the presence of lactate. Scale bar = 50 µm. Data are shown as mean ± SEM; comparisons were made by two-tailed *t*-test, n = 3-6. **p < 0.01.

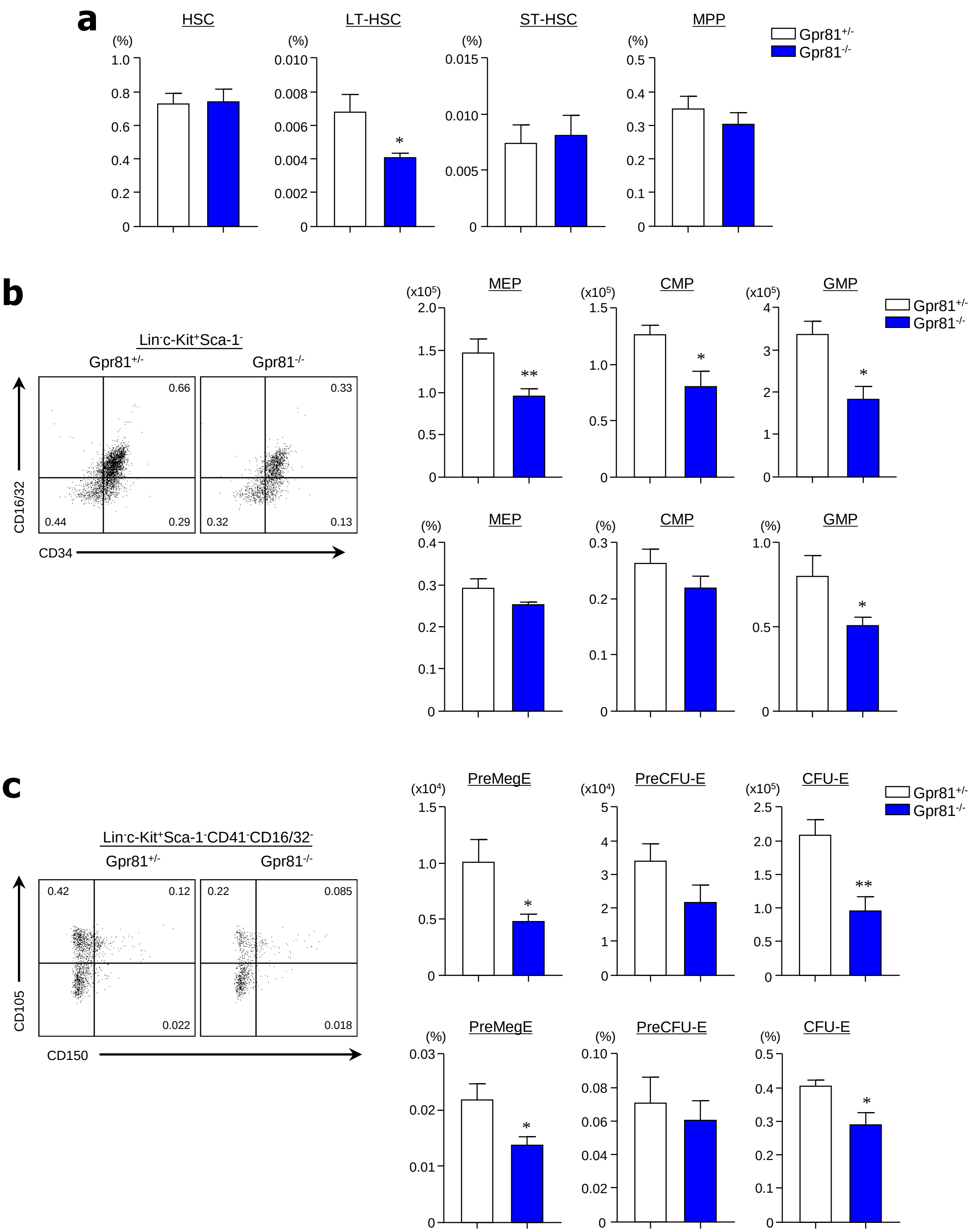

Supplementary Figure. 3 Lactate-Gpr81 signals play an indispensable role in BM reconstitution after injury. FACS analysis in total BM cells of $Gpr81^{+/-}$ and $Gpr81^{-/-}$ mice at 1 week after 6 Gy irradiation. (**a**) Percentages of HSCs, LT-HSCs, ST-HSCs, and MPPs in total BM cells. (**b**) Representative FACS plots for percentages and absolute cell numbers of MEPs, CMPs, and GMPs in total BM cells. (**c**) Representative FACS plots for percentages and absolute cell numbers of PreMegEs, PreCFU-Es, and CFU-Es in total BM cells. CD150 antibody (clone TC15-12F12.2) was used for FACS analysis of A, B, and C. Data are means ± SEM; comparisons were made by two-tailed *t*-test, n = 3-5. *$p < 0.05$, **$p < 0.01$. Data were combined from ≥ 2 independent experiments.

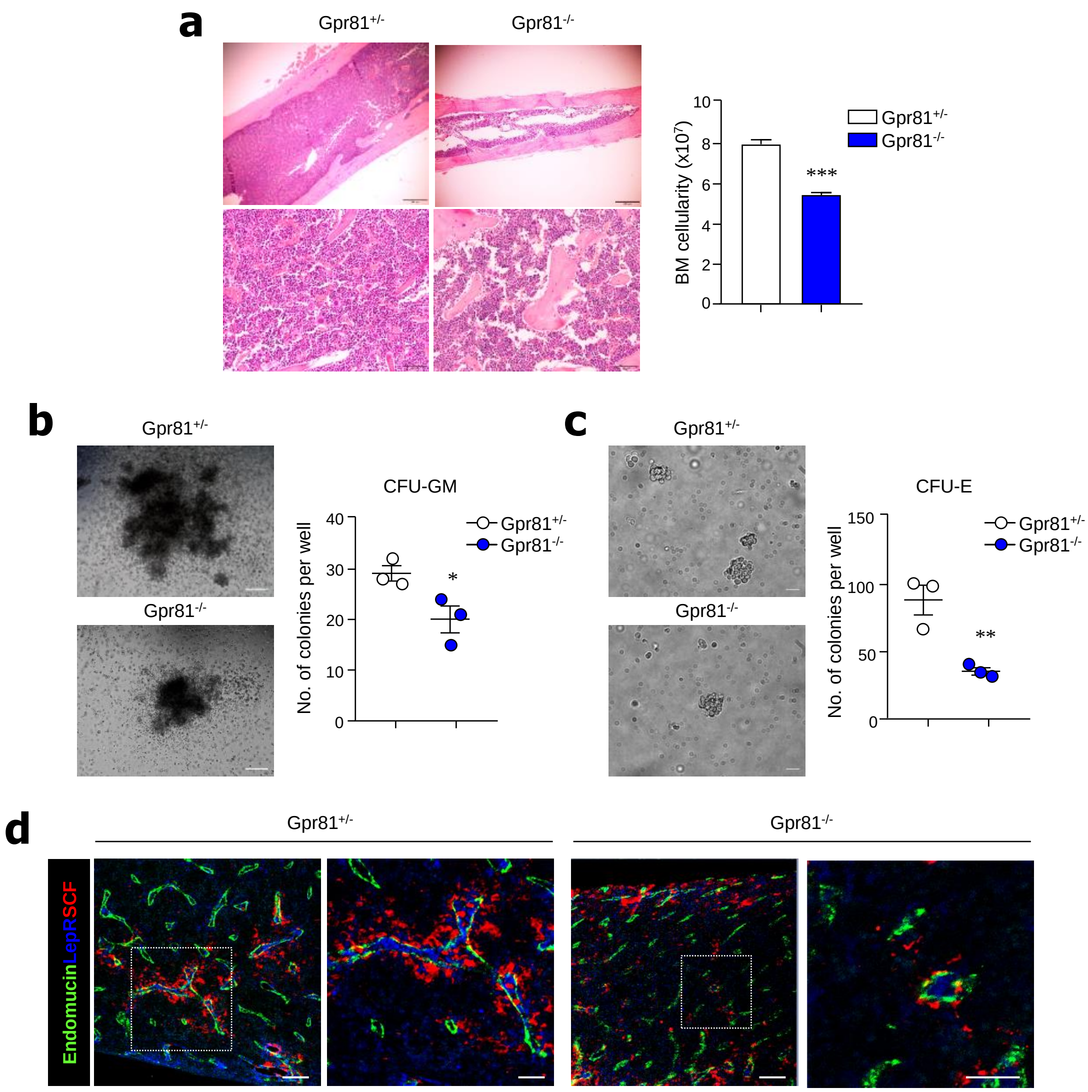

Supplementary Figure. 4 Lactate-Gpr81 signals play an indispensable role in BM reconstitution after injury. Analysis of BM cells of Gpr81$^{+/-}$ and Gpr81$^{-/-}$ mice at 1 week after busulfan treatment. (**a**) Representative BM tissue section stained with H&E. (**b**) CFU-GM assay using flushed total BM cells. Scale bar = 200 μm. (**c**) CFU-erythroid (CFU-E) assay using flushed total BM cells. Scale bar = 20 μm. (**d**) Immunohistochemical analysis for endomucin (green), LepR (blue), and SCF (red) expression in BM tissues of mice. Scale bar = 50 μm (left) and = 20 μm (right). Data are mean ± SEM; comparisons were made by two-tailed *t*-test, n = 3. *p < 0.05 , **p < 0.01, ***p < 0.001. Data were combined from two independent experiments.

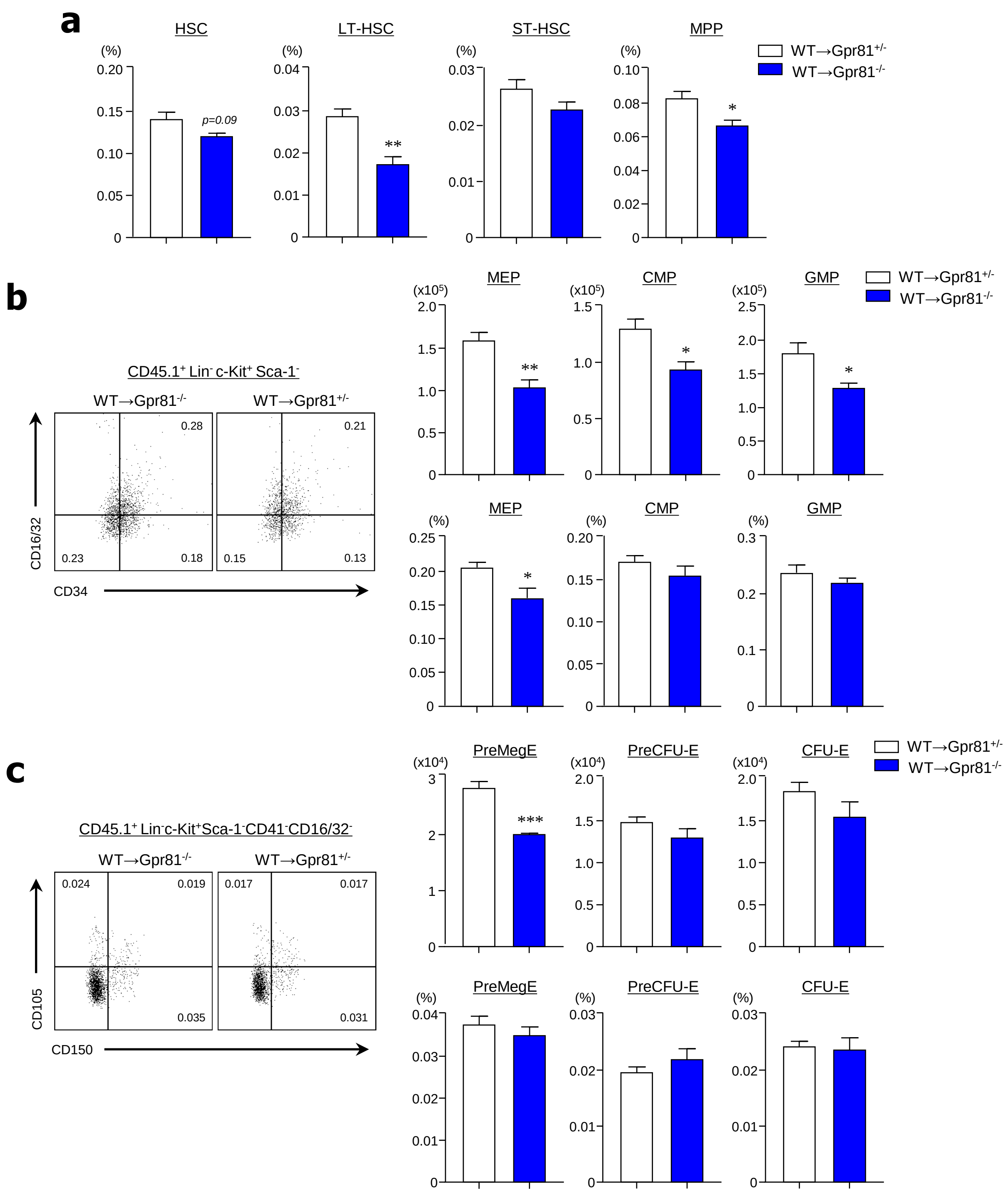

Supplementary Figure. 5 HSC proliferation was regulated by the BM environment in a Gpr81-dependent manner. FACS analysis in femurs and tibias of recipient mice 3 months after BMT. (**a**) Percentages of HSCs, LT-HSCs, ST-HSCs, and MPPs in total BM cells. (**b**) Representative FACS plots for percentages and absolute cell numbers of MEPs, CMPs, and GMPs in total BM cells. (**c**) Representative FACS plots for percentages and absolute cell numbers of PreMegEs, PreCFU-Es, and CFU-Es in total BM cells. CD150 antibody (clone TC15-12F12.2) was used for FACS analysis of A and C. Data are mean ± SEM; comparisons were made by a two-tailed *t*-test, n = 5. *p < 0.05 , **p < 0.01, ***p < 0.001. Data were repeated twice in independent experiments.

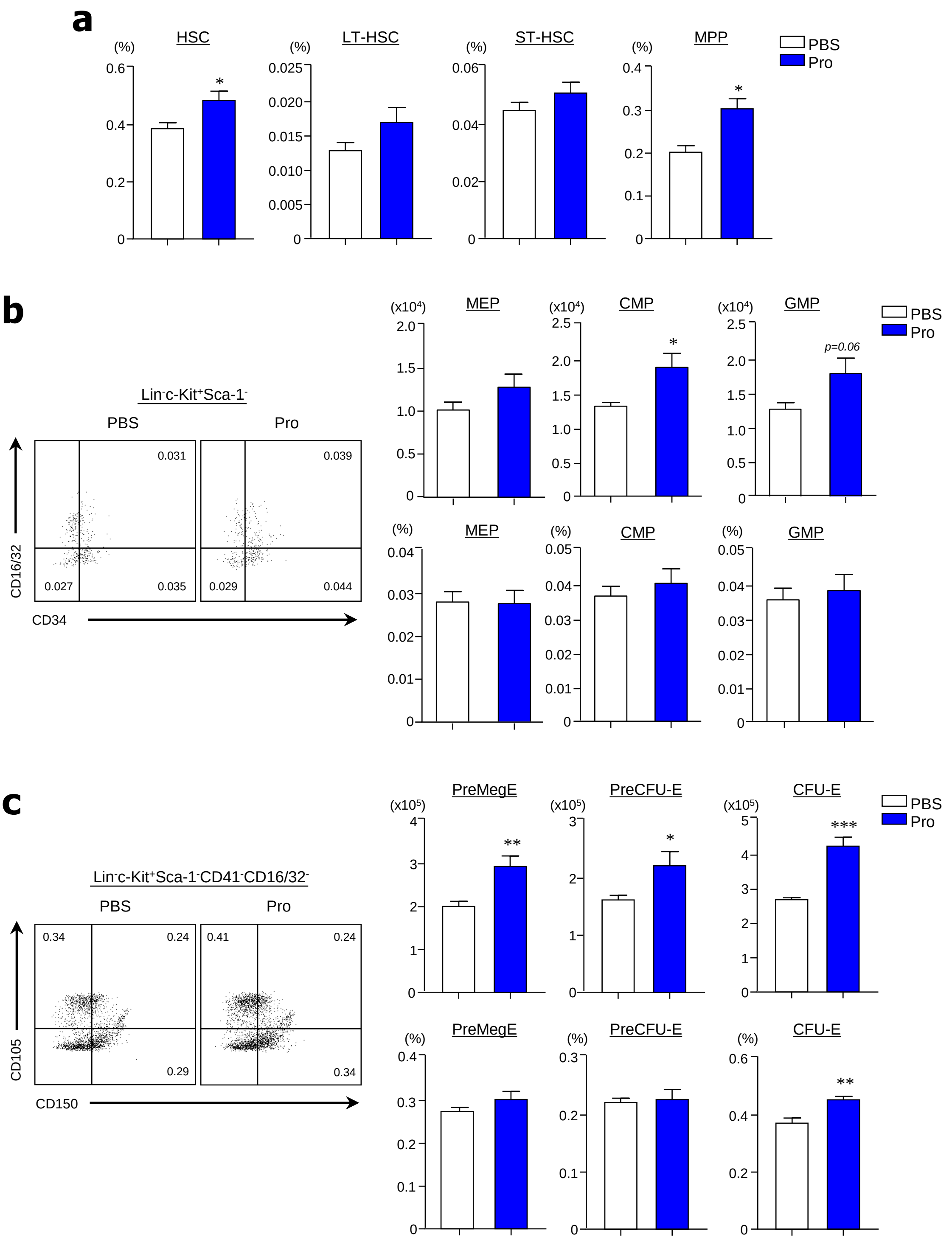

Supplementary Figure. 6 LAB-rich probiotics regulate differentiation of erythrocyte-lineages. FACS analysis in BM of C57BL/6 mice fed probiotics (Pro; VSL#3) or vehicle for 1 week. (**a**) Percentage of HSCs, LT-HSCs, ST-HSCs, and MPPs in total BM cells. (**b**) Representative FACS plots for percentages and absolute cell numbers of MEPs, CMPs, and GMPs in total BM cells. (**c**) Representative FACS plots for percentages and absolute cell numbers of PreMegEs, PreCFU-Es, and CFU-Es in total BM cells. Data are shown as mean ± SEM; comparisons were made by two-tailed *t*-test, n = 5. *p < 0.05 , **p < 0.01, ***p < 0.001.

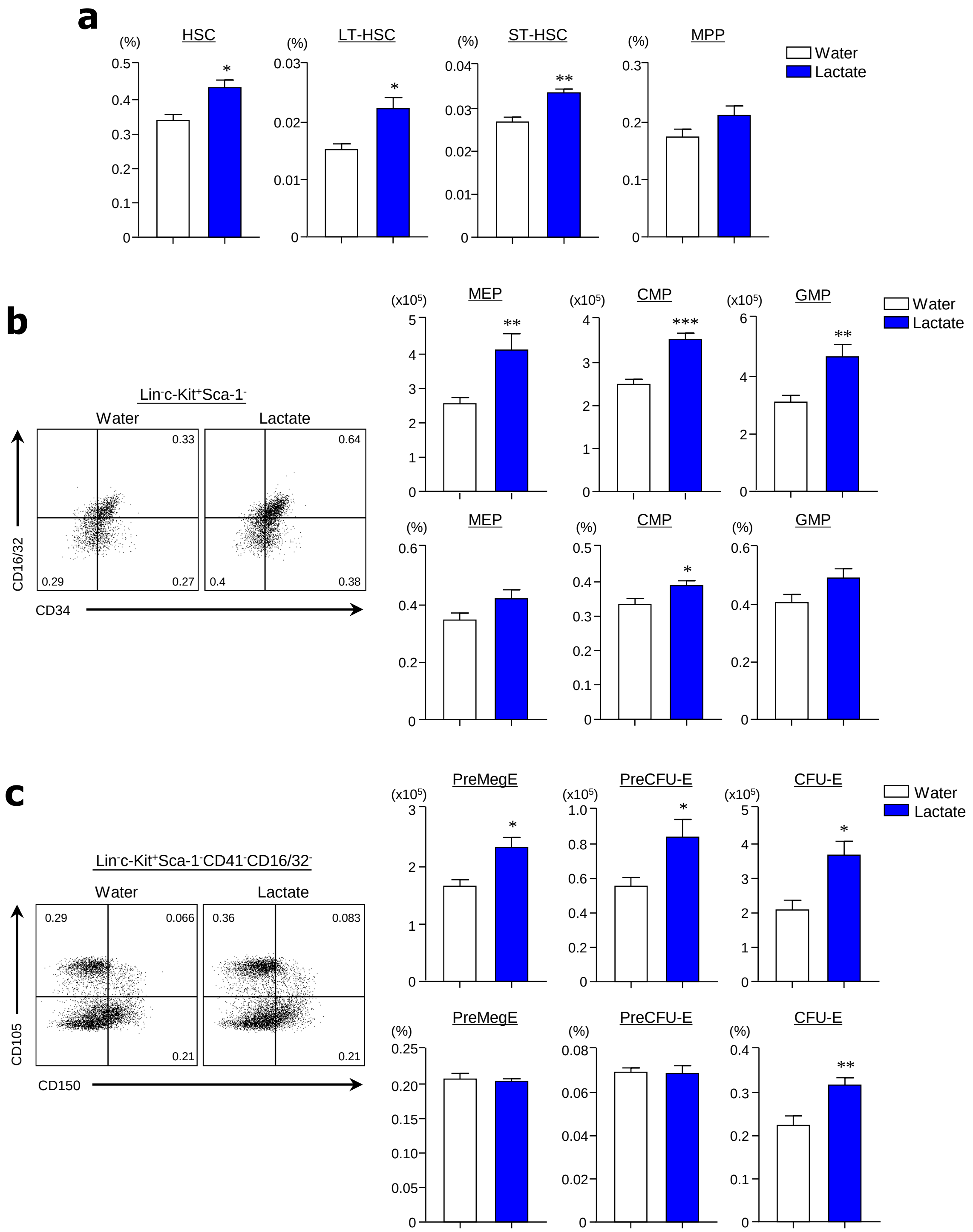

Supplementary Figure. 7 Lactate induces proliferation of HSCs in erythrocyte-lineages. FACS analysis in BM of C57BL/6 mice given drinking water supplemented with lactate (10 mM) for 1 week. (**a**) Percentage of HSCs, LT-HSCs, ST-HSCs, and MPPs in total BM cells. (**b**) Representative FACS plots for percentages and absolute cell numbers of MEPs, CMPs, and GMPs in total BM cells. (**c**) Representative FACS plots for percentages and absolute cell numbers of PreMegEs, PreCFU-Es, and CFU-Es in total BM cells. Data are shown as mean ± SEM; comparisons were made by two-tailed *t*-test, n = 3-5. *$p < 0.05$, **$p < 0.01$, ***$p < 0.001$. Data were combined from two independent experiments.

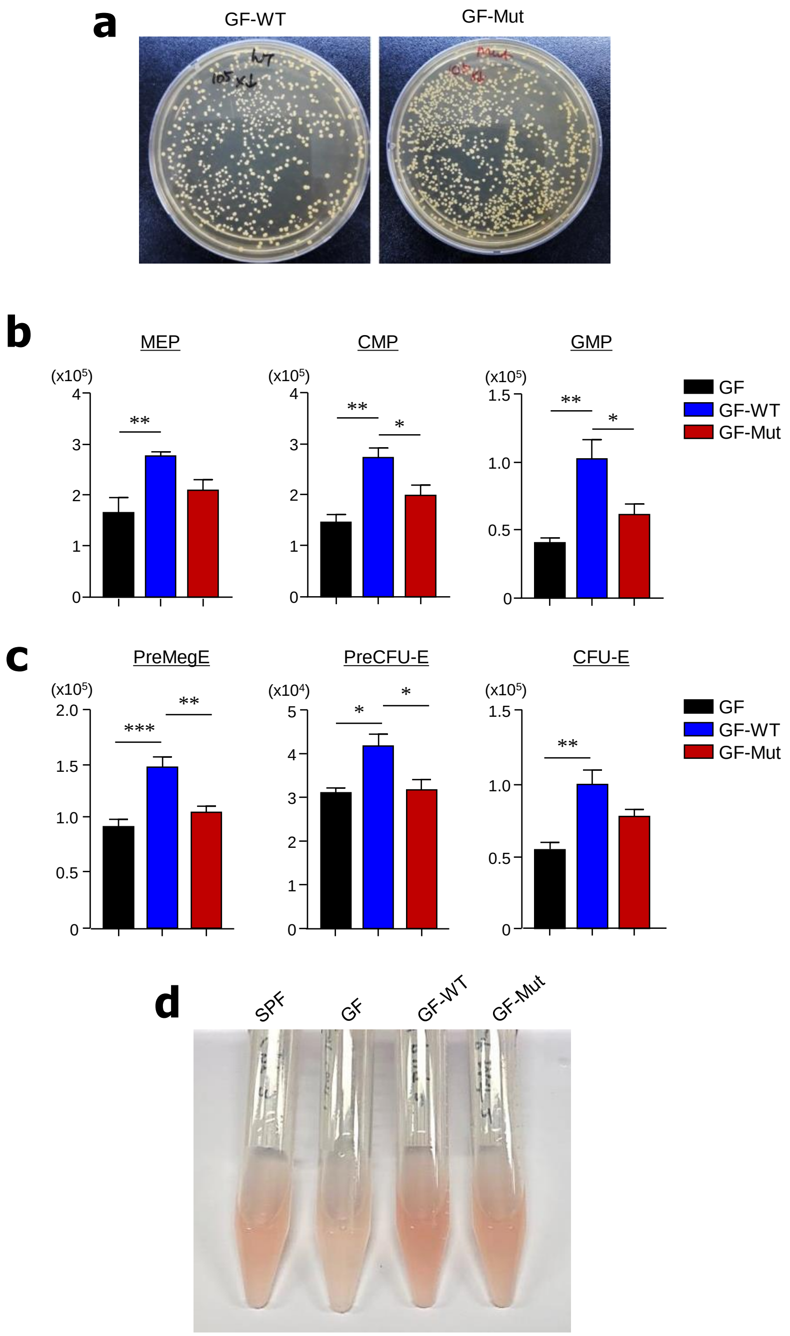

Supplementary Figure. 8 Oral administration of *Lactobacillus plantarum* enhances self-renewal of HSCs. Wild-type (WT) *L. plantarum* or *L. plantarum* Δ*ldhD*Δ*ldhL* mutant (Mut) strains were orally administered to GF mice once and analyzed at 16 weeks. (**a**) Representative CFU images of mouse feces. (**b**) Absolute cell numbers of MEPs, CMPs, and GMPs in total BM cells. (**c**) FACS analysis of absolute cell numbers of PreMegEs, PreCFU-Es, and CFU-Es in total BM cells. CD150 antibody (clone TC15-12F12.2) was used for FACS analysis. (**d**) Representative image of BM cell suspensions. Each group was suspended with an equal amount of PBS. Data are shown as mean ± SEM; comparisons were made by two-tailed *t*-test, n = 3-5. *p < 0.05 , **p < 0.01, ***p < 0.001. Data were repeated twice in independent experiments.
